# Gastrointestinal Lesion Detection Using Ensemble Deep Learning Through Global Contextual Information

**DOI:** 10.3390/bioengineering12121329

**Published:** 2025-12-05

**Authors:** Vikrant Aadiwal, Vishesh Tanwar, Bhisham Sharma, Dhirendra Prasad Yadav

**Affiliations:** 1 Chitkara University Institute of Engineering and Technology, Chitkara University, Rajpura 140401, Punjab, India; 2 Department of Computer Engineering & Applications, GLA University, Mathura 281406, Uttar Pradesh, India

**Keywords:** gastrointestinal, medical images, disease, detection, ensemble, deep learning, attention

## Abstract

The presence of subtle mucosal abnormalities makes small bowel Crohn’s disease (SBCD) and other gastrointestinal lesions difficult to detect, as these features are often very subtle and can closely resemble other disorders. Although the Kvasir and Esophageal Endoscopy datasets offer high-quality visual representations of various parts of the GI tract, their manual interpretation and analysis by clinicians remain labor-intensive, time-consuming, and prone to subjective variability. To address this, we propose a generalizable ensemble deep learning framework for gastrointestinal lesion detection, capable of identifying pathological patterns such as ulcers, polyps, and esophagitis that visually resemble SBCD-associated abnormalities. Further, the classical convolutional neural network (CNN) extracts shallow high-dimensional features; due to this, it may miss the edges and complex patterns of the gastrointestinal lesions. To mitigate these limitations, this study introduces a deep learning ensemble framework that combines the strengths of EfficientNetB5, MobileNetV2, and multi-head self-attention (MHSA). EfficientNetB5 extracts detailed hierarchical features that help distinguish fine-grained mucosal structures, while MobileNetV2 enhances spatial representation with low computational overhead. The MHSA module further improves the model’s global correlation of the spatial features. We evaluated the model on two publicly available DBE datasets and compared the results with four state-of-the-art methods. Our model achieved classification accuracies of 99.25% and 98.86% on the Kvasir and Kaither datasets.

## 1. Introduction

Inflammatory bowel disease (IBD) is a chronic, non-infectious inflammatory condition of the gastrointestinal system, mainly including Crohn’s disease, ulcerative colitis, polyps, and esophagitis. Crohn’s disease is notable as it can impact any segment of the gastrointestinal tract, frequently affecting the small intestine, and is linked to symptoms like stomach pain, diarrhea, weight loss, and perianal problems [1]. The small intestine has always been regarded as the “black box” of the gastrointestinal system because of its restricted accessibility via traditional endoscopy. Despite capsule endoscopy enabling comprehensive visualization, its dependence on extensive image data presents obstacles for prompt and precise diagnosis. These constraints are particularly significant in SBCD, where delayed diagnosis may result in severe consequences [2]. Gastrointestinal illnesses constitute a significant health issue globally. Colonoscopy is the conventional diagnostic technique for colon diseases that requires significant time and costs. One of the most severe signs of gastrointestinal illnesses is bleeding, which can happen in both the upper and lower parts of the tract. Up to 100 to 200 cases per 100,000 people worldwide occur annually due to upper gastrointestinal bleeding alone, and these can be of a potentially fatal nature. Within this spectrum, SBCD poses a particular risk due to the chronic inflammatory course that offers complications such as malnutrition, obstruction, and increased healthcare burden. Traditional diagnostic methods mainly consist of endoscopic procedures, imaging techniques, and laboratory tests. Among those, colonoscopy and endoscopy are the most widely used approaches, enabling a direct view of the gastrointestinal tract for bleeding, ulcers, or tumors, but they are invasive, time-consuming, and often uncomfortable for the patients. CT scans, MRIs, and ultrasounds are the noninvasive imaging modalities; however, due to their high cost, limited accessibility, and lower sensitivity in detecting early-stage diseases, they become inadequate [3,4]. Laboratory-based methods, including fecal occult blood tests and stool cultures, help in diagnosing hidden bleeding or infections, although they are often inaccurate and non-specific if conducted solely. Although these traditional techniques are still the gold standard in clinical practice, their drawbacks indicate the need for more efficient, accurate, and less invasive diagnostic solutions [5,6].

To address the abovementioned limitations, it is important to note that the proposed framework is designed as a generic gastrointestinal lesion detection system, rather than a disease-specific diagnostic model. Although small-bowel Crohn’s disease (SBCD) served as the motivating application, the current experiments employ publicly available colon and esophageal image datasets (Kvasir and Kaggle) due to the limited accessibility of open DBE datasets. These datasets provide visually analogous patterns such as ulcers, mucosal inflammation, and polyps that serve as reliable surrogates for evaluating the model’s ability to generalize across gastrointestinal pathologies. Our model enhanced spatial context and classification accuracy by combining the strong feature-extracting powers of EfficientNetB5 and MobileNetV2 with an MHSA method. EfficientNetB5 extracts detailed hierarchical features that help distinguish fine-grained mucosal structures, while MobileNetV2 enhances spatial representation with low computational overhead. The MHSA module further improves the model’s global correlation of the spatial features.

The notable contributions of the manuscript are as follows:

- EfficientNetB5 is a strong convolutional backbone, and it is used for hierarchical feature extraction. The EfficientNetB5 extracts high-dimensional local fine-grained textures and boundaries that help it to determine the slight morphological deformities and ulcerations typical of small-bowel Crohn’s disease. In conjunction with this, MobileNetV2 offers an effective spatial coding that is lightweight, hence ensuring that vital mucosal details that are essential in identifying GI disease are not lost at the cost of high computational demands.
- We ensemble the features of the EfficientNetB5 and MobileNetV2 and feed them into a multi-head self-attention (MHSA) mechanism to extract the global context of the spatial feature. This helps the model focus on the most important areas related to ulcers while still keeping the important small details.
- We evaluated our proposed model on two datasets and compared it with other state-of-the-art models.

The rest of the paper is organized as follows: Section 2 reviews prior research on Crohn’s disease detection using endoscopy and deep learning. Section 3 describes the proposed methodology, including architectural components and training strategy. Section 4 presents the results and performance metrics based on extensive experimentation. Section 5 is the discussion section, and finally, Section 6 concludes the paper.

## 2. Related Work

Kumar et al. [3] applied EfficientNetB0, ResNet50, and InceptionV3 for GI bleeding detection from wireless capsule endoscopy (WCE) images. An accuracy as high as 98.4% was attained by their proposed CNN, outperforming the conventional approach of SIFT with SVM. It proved very effective in the detection of slight bleeding patterns. EfficientNetB0 showed an excellent trade-off between accuracy and computational efficiency. Alhajlah et al. [7] conducted a study focused on overcoming the difficulty of distinguishing between infected and healthy gastrointestinal regions, a key factor limiting precise classification. They utilized Mask R-CNN combined with fine-tuned ResNet50 and ResNet152 for feature extraction. The detected regions of interest were used for training via transfer learning, and features from both models were fused using a serial approach. An improved ant colony optimization algorithm was employed for optimal feature selection, and the selected features were classified using machine learning classifiers. Experiments on a public dataset achieved an accuracy of 96.43%. Sivari et al. [8] proposed a deep learning-based hybrid bi-level stacking ensemble for accurate and objective gastrointestinal findings classification. At the first level, three novel CNN models were trained using 5-fold cross-validation, and their predictions were combined. A second-level machine learning classifier then processed these predictions to produce the final classification. Experimental evaluation on the KvasirV2 and HyperKvasir datasets achieved accuracies of 98.42% and 98.53%, respectively, with corresponding MCCs (Matthews correlation coefficients) of 98.19% and 98.39%. Thomas et al. [9] proposed a transfer learning-based method using various pretrained models for the identification and classification of digestive diseases, which can affect organs such as the stomach, intestines, liver, pancreas, and gallbladder. They compared multiple architectures, achieving notable improvements in accuracy, precision, and recall over existing methods. Among the evaluated models, EfficientNetB0 yielded the highest performance with 98.01% accuracy, 98% precision, and 98% recall, demonstrating its effectiveness in digestive disease classification.

For early BE (Barrett’s esophagus) neoplasia detection, Kusters et al. [10] introduced a CADe system that was optimized through systematic evaluation of 10 backbone architectures, 4 segmentation decoders, domain-specific pretraining, data augmentation, multi-expert labeling, and inference fusion methods. A hybrid CNN transformer with domain-specific pretraining delivered the best results, achieving up to 12.8% improvement over the second-best state-of-the-art system across 9 test sets, with statistically significant gains (*p* = 0.0019–0.031) in classification and localization accuracy. A recent study done by Sharma et al. [11] addressed the limitations of manual gastrointestinal disease diagnosis from colonoscopy and wireless capsule endoscopy images by developing automated deep learning models. Using the KVASIR benchmark dataset, which includes images of polyps, ulcerative colitis, esophagitis, and healthy colon tissue, multiple CNN architectures, including a baseline model and transfer learning with VGG16, InceptionV3, and ResNet50, were trained with n-fold cross-validation and data augmentation. The ResNet50-based transfer learning model achieved the highest performance, with 99.80% training accuracy (precision 100%, recall ~99%), 99.50% validation accuracy, and 99.16% accuracy on a 1200-image test set. These results demonstrate that ResNet50 significantly outperforms existing GI disease classification systems in both accuracy and robustness.

Ramzan et al. [12] developed a CADx system for GI tract disease diagnosis using fused features from LAB color-space preprocessing, local binary patterns, and deep learning models (InceptionNet, ResNet50, and VGG-16). PCA, entropy, and mRMR were tested for optimal feature selection, followed by training various classifiers. Evaluated on KVASIR, NERTHUS, and stomach ulcer datasets, the subspace discriminant classifier achieved 95.02% accuracy on KVASIR, outperforming existing state-of-the-art methods. A study done by Wei et al. [13] introduced a multi-granularity labelled upper gastrointestinal endoscopy image dataset and proposed a multi-granularity collaborative classification network (MGCN) that emulates physicians’ coarse-to-fine diagnostic process. The framework employs a weakly supervised multi-granularity self-attention module for feature extraction and a collaborative loss module for classification, leveraging inter-granularity correlations. On the proposed dataset, MGCN achieved state-of-the-art performance with 85.73% fine-grained, 93.49% medium-grained, and 99.01% coarse-grained accuracy, surpassing HRN, PMG, and SnapMix.

Fan et al. [14] proposed a fault-tolerant automatic localization system for pylorus and ileocecal valve boundaries in wireless capsule endoscopy (WCE), integrating a ResNet50-based gastrointestinal classification model with a sliding pin algorithm. The model classified WCE images into three gastrointestinal regions with an average accuracy of 92.21% (recognition rate 88–96%) on 108,542 images, while the sliding pin algorithm corrected misclassifications. Validation on 31 independent WCE cases achieved mean locating errors of 26 frames for the pylorus and 65 frames for the ileocecal valve, outperforming existing state-of-the-art methods.

Babu et al. [15] proposed a Deep Hexa model combined with ShuffleNet statistical features, UNet segmentation features, and capsule network classification. Achieved 99.38% accuracy, surpassing Duo-deep (+0.25%), ESKNN (+2.89%), VGG-16 (+3.06%), Inception-ResNet-v2 (+0.90%), and Fusion network (+1.39%). The DCDS-Net model, proposed by Asif et al. [16] based on densely connected depthwise separable convolutions with residual connections and dense blocks, was developed for accurate diagnosis of GI diseases from endoscopic images. The architecture integrates global average pooling, batch normalization, dropout, and block-wise fine-tuning via transfer learning to address limited labeled medical data. Evaluated on 6000 labeled endoscopic images (4 GI disease classes) with data augmentation, the model achieved 99.33% accuracy, 99.37% precision, and 99.32% recall, outperforming all pretrained and existing models. Grad-CAM visualizations confirmed disease-relevant regions, demonstrating high potential for assisting endoscopists in automated diagnosis. Table 1 shows the summary of the related work.

## 3. Methodology

**Figure 1:**
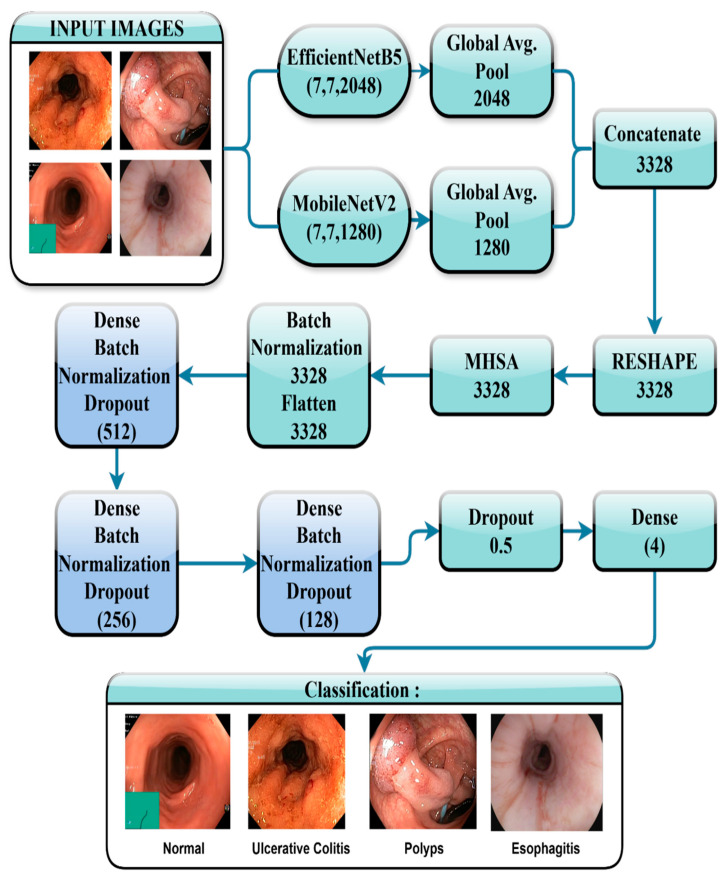
Architecture of the proposed model.

This part highlights the methods utilized to augment classification performance through the concatenation of feature maps from EfficientNetB5 and MobileNetV2. The proposed approach utilizes the synergistic advantages of two CNN models to derive comprehensive spatial and semantic properties. EfficientNetB5, recognized for its depthwise convolution and squeeze and excitation methods, acquires high-level feature representations, whereas MobileNetV2 offers lightweight yet effective feature extraction through its inverted residuals. The extracted feature maps are fused together to provide a richer and more complete representation, which is followed by a classification layer that will refine the final predictions. This approach keeps an effective balance between accuracy and computational cost, therefore making it well-suited for real-world usage. Figure 1 shows a graphical representation of our proposed model.

### 3.1. EfficientNetB5

EfficientNet models introduce a novel scaling method called compound scaling, which systematically balances three key dimensions of a neural network: depth (number of layers), width (number of channels per layer), and resolution (input image size). Unlike conventional approaches that scale only one of these dimensions at a time, EfficientNet simultaneously scales all three in a structured manner, allowing it to achieve better accuracy with fewer parameters and lower computational costs. EfficientNetB5 is a better image recognition model than its predecessor, EfficientNetB0, because it is bigger but still works well. The MBConv (mobile inverted bottleneck convolution) is the core part of the system. It is based on the layers used in MobileNetV2. Each MBConv block comprises a few phases. First, it adds more channels to capture more features. Then, it uses a unique type of convolution called depthwise convolution to cut down on processing. It also has a squeeze and excitation function that helps the model pay more attention to essential features by taking the whole image into view; this shifts what the model pays attention to in order to allow it to pick out important facts. The model starts with a simple convolution layer whose job is to prepare the input picture. Then, there are several layers of MBConv blocks. These stages gradually reduce the image’s size while raising the level of detail and complexity of the features, thereby making the network understand images more effectively. The number of blocks and the width multiplier are decided based on the compound scaling formula. The deeper layers pull out more abstract and high-level semantic information, while the early layers focus on low-level edge and texture details. The final convolutional layers are followed by a global average pooling that aggregates spatially dispersed information into a compact feature representation; a fully connected layer then maps those features to the output classes.

**Figure 2:**
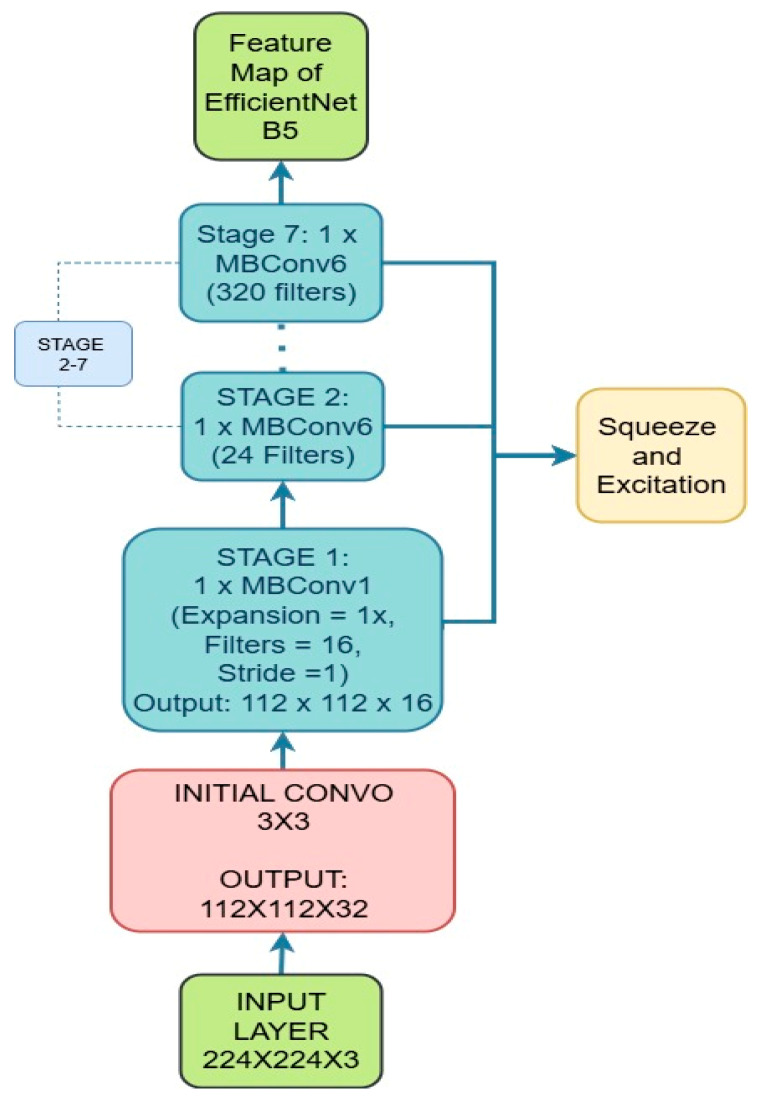
EfficientNetB5 architecture.

EfficientNetB5 begins with a stem layer that processes the input image. The input X is an image of size 256 × 256 × 3. The stem applies a 3 × 3 convolution to capture low-level features. This convolution utilizes a set of filters *W*_0_, a kernel size *k* = 3, a stride *s* = 2 (which halves the spatial dimensions), and padding *p* = 1 to maintain alignment. Mathematically, this is represented in Equation (1).

(1) $$F_{m}=Conv\left(X\right)$$

where *F_m_* represents the feature maps obtained after the initial convolution. This step is crucial, as it extracts essential edge and texture features before passing the data through deeper layers of the network. Following the stem, EfficientNetB5 is constructed from several MBConv blocks (Mobile Inverted Bottleneck Convolution blocks). Each MBConv block is a combination of multiple operations that work together to efficiently extract complex features. These blocks include the following steps, as shown in Figure 2.

#### 3.1.1. Depthwise Separable Convolution

Instead of a full convolution that mixes channels and spatial information simultaneously, a depthwise convolution applies a separate filter to each channel. For the *i*th block, this operation is given by Equation (2).

(2) $$F_{i}=DepthwiseConv\left(F_{m-1\;},W_{i},\;k=3,s,p\right)$$

where *F_m_*_−1_ is the input feature map from the previous layer, *W_i_* represents the depthwise convolution filters, *k* is the kernel size, commonly 3, *s* is the stride, which might vary depending on whether the spatial resolution should be reduced, and *p* is the padding.

#### 3.1.2. Squeeze and Excitation (SE)

The SE block recalibrates channel-wise feature responses by explicitly modeling interdependencies between channels. First, a global average pooling is applied to *F_i_* to squeeze global spatial information into a channel descriptor represented by Equation (3).

(3) $$S=GlobalAvgPool\left(F_{i}\right)$$

Then, this descriptor is passed through a small fully connected network with a non-linearity, typically a sigmoid, represented by σ, to generate scaling factors shown in Equation (4).

(4) $$S_{i}=\sigma \left(W_{SE}\cdot S\right)\;$$

where *W_SE_* are the weights of the Squeeze-and-Excitation (SE) block. These scaling factors *S_i_* are then used to excite (i.e., reweight) the original feature map *F_i_*, effectively emphasizing informative features and suppressing less useful ones, as shown by Equation (5).

(5) $$F_{map}=F_{i}+S_{i}$$

This residual connection helps with gradient flow and stabilizes training.

### 3.2. MobileNetV2

MobileNetV2 is an efficient convolutional neural network architecture specifically designed for mobile and embedded applications. It builds upon the concept of depthwise separable convolutions introduced in MobileNetV1 and introduces a novel building block known as the inverted residual block with a linear bottleneck. This design principle allows the network to maintain high accuracy while significantly reducing computational complexity and memory usage. The first layer of MobileNetV2 is a normal convolution layer that processes the input image, which is usually 224 × 224 × 3 pixels, to make a set of feature maps with more channel depth. This first convolution utilizes a 3 × 3 kernel with a stride of 2 to make the spatial dimensions smaller while still collecting early visual information. This layer’s output is the input for a sequence of inverted residual blocks. The first step in each inverted residual block is an expansion phase, in which a 1 × 1 convolution projects the input tensor into a space with more dimensions. If the input tensor has t channels, the enlarged tensor will have 6t channels. The expansion factor is commonly set to 6. This step is very important since it gives the next depthwise convolution more space. Each inverted residual block starts with an expansion phase, where the input tensor is projected into a higher-dimensional space using a 1 × 1 convolution. The expansion factor is usually set to 6, meaning that if the input tensor has t channels, the expanded tensor will have 6t channels. This phase is crucial for providing sufficient capacity for the subsequent depthwise convolution.

Once the channels are expanded, a depthwise convolution is applied. In this operation, a single convolutional filter is applied to each input channel separately. This significantly reduces the number of computations compared to a standard convolution that mixes channels. The depthwise convolution normally utilizes a 3 × 3 kernel, and then it is followed by batch normalization and a non-linear activation (commonly ReLU6). This adds non-linearity while keeping the computation efficient. After this, the linear bottleneck phase ends the block. A 1 × 1 convolution is employed in this step to project the features back down to a lower-dimensional space that matches the original number of channels or a desired lower dimensionality. It is important to note that no non-linearity is used after this projection. The linearity keeps the manifold structure of the features, which makes sure that important information is not lost. When the spatial dimensions and channel depths of the input and output of an inverted residual block are the same, a shortcut connection is employed. This residual connection adds the original input tensor directly to the output of the block, improving gradient flow during training and further stabilizing the learning process. The combination of these operations—expansion, depthwise convolution, linear bottleneck, and residual connection—constitutes the core of MobileNetV2′s design, allowing it to achieve an excellent trade-off between performance and efficiency.

**Figure 3:**
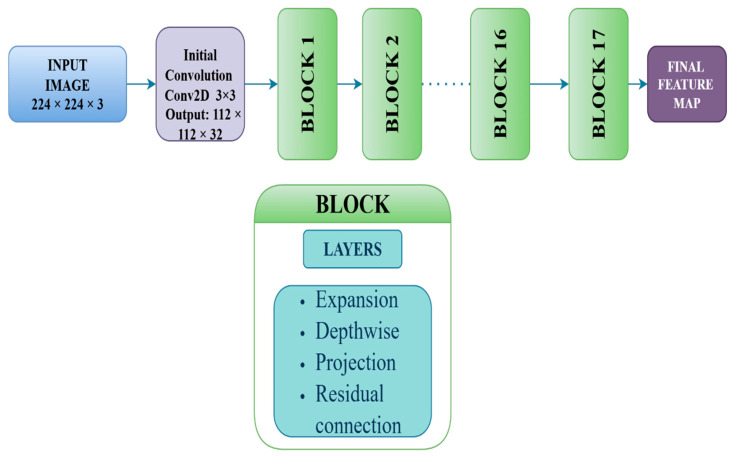
Architecture of MobileNetV2.

The overall architecture of MobileNetV2 is built by stacking multiple inverted residual blocks with varying configurations. Figure 3 presents the MobileNet V2 framework adopted in the proposed methodology. After the initial convolutional layer, the network employs a series of blocks that progressively reduce the spatial dimensions while increasing the channel depth. This progression results in a final feature representation that is compact yet rich in information. Before the final classification layers, a global average pooling operation aggregates the spatial information across the entire feature map, and a final fully connected layer (or a 1 × 1 convolution in fully convolutional implementations) maps the pooled features to the output classes.

The initial convolution layer has a convolution size of 112 × 112 and a filter size to extract the spatial features from the image. The initial convolution operation is mathematically defined as follows:

(6) $$F_{w}=\;ReLu(W_{0}.X_{n})$$

(7) $$\;F_{d}=ReLu(W_{0}.F_{w})$$

where *X_n_* is the input image, *W*_0_ are the convolutional weights, *k* = 3 is the kernel size, and the stride is 2. After that, the projection reduces the number of channels back to a lower-dimensional space without applying a non-linearity, as shown in Equation (8).

(8) $$F_{proj}=Conv2D\left(F_{p},W_{proj},\;k=1,s=1,p=0\right)$$

where $W_{proj}$ values are the projection weights. Finally, the inverted residual connection is applied to match input and output dimensions. The residual connection is applied as follows:

(9) $$F_{out}=F_{in}+F_{proj}$$

### 3.3. Global Average Pooling 2D in EfficientNet and MobileNetV2

Global average pooling (GAP) is a crucial operation in modern deep learning architecture, particularly for reducing spatial dimensions while preserving the most important feature representation. It operates by averaging the spatial dimensions (height and width) of each feature map, resulting in a single scalar per channel. Mathematically, it can be defined in Equation (10).

(10) $$EficientNetB5=GAP(F_{map}),\;MobileNetV2=GAP\left(F_{out}\right)$$

In EfficientNet, the feature map before GAP has a shape of (None, 7, 7, and 2048). The GAP operation reduces the spatial dimension to (None, 2048) by averaging over the 7 × 7 spatial dimensions. This serves as the final compressed feature representation before the classification head.

Similarly, on MobileNetV2, the feature map before GPA has a shape of (None, 7, 7, 1280). The GPA operation averages each channel’s spatial region, resulting in an output shape of (None, 1280). This compact representation is then passed to the classifier. Both architectures use global average pooling to significantly reduce the number of parameters, improve generalization, and pre-learn features for the final classification layer efficiently.

### 3.4. Concatenation of Feature Maps

The proposed approach combines feature maps of EfficientNetB5 and MobileNetV2 to create a unified and augmented representation of features. The model combines these feature vectors and thus takes advantage of the strengths of the two architectures to result in better performance of classification. Global average pooling (GAP) is performed on the last feature maps of each network and concatenates the results together so that feature fusion is efficient. EfficientNetB5 results in a flat global average pooling output of shape (None, 2048) and a MobileNetV2 output of shape (None, 1280).

The feature concatenation process involves combining these feature maps along the feature dimension using a concatenation layer as shown in Equation (11).

(11) $$F_{concat}=Concatenate(F_{EfficientNetB5},F_{MobileNetV2})$$

In Equation (11), $F_{EfficientNetB5}\in R^{2048}$ represents the extracted feature vector from EfficientNetB5, and $F_{MobileNetV2}\in R^{1280}$ represents the extracted feature vector from MobileNetV2. The resulting concatenated feature vector F_concat_ has an output shape of (None, 3328). The purpose of this step is to make sure that the final classification decision will be made with the help of both high-level abstract features of EfficientNetB5 and lightweight and efficient features of the MobileNetV2.

Having concatenated the feature vectors of EfficientNetB5 and MobileNetV2, the final feature map will have an output shape of (None, 3328). A reshaping operation is then used to further refine the feature representation and ready it to be processed further.

The reshaping process changes the combined feature map by adding a new dimension, resulting in an output shape like (None, 1, 3328) as shown in Equation (12).

(12) $$F_{reshaped}=Reshape(F_{concat})$$

In Equation (12), $F_{concat}\in R^{3328}$ represents the concatenated feature vector, and $F_{reshaped}\in R1\times 3328\;$ represents the reshaped feature map with an additional spatial dimension.

### 3.5. Multi-Head Self-Attention (MHSA)

**Figure 4:**
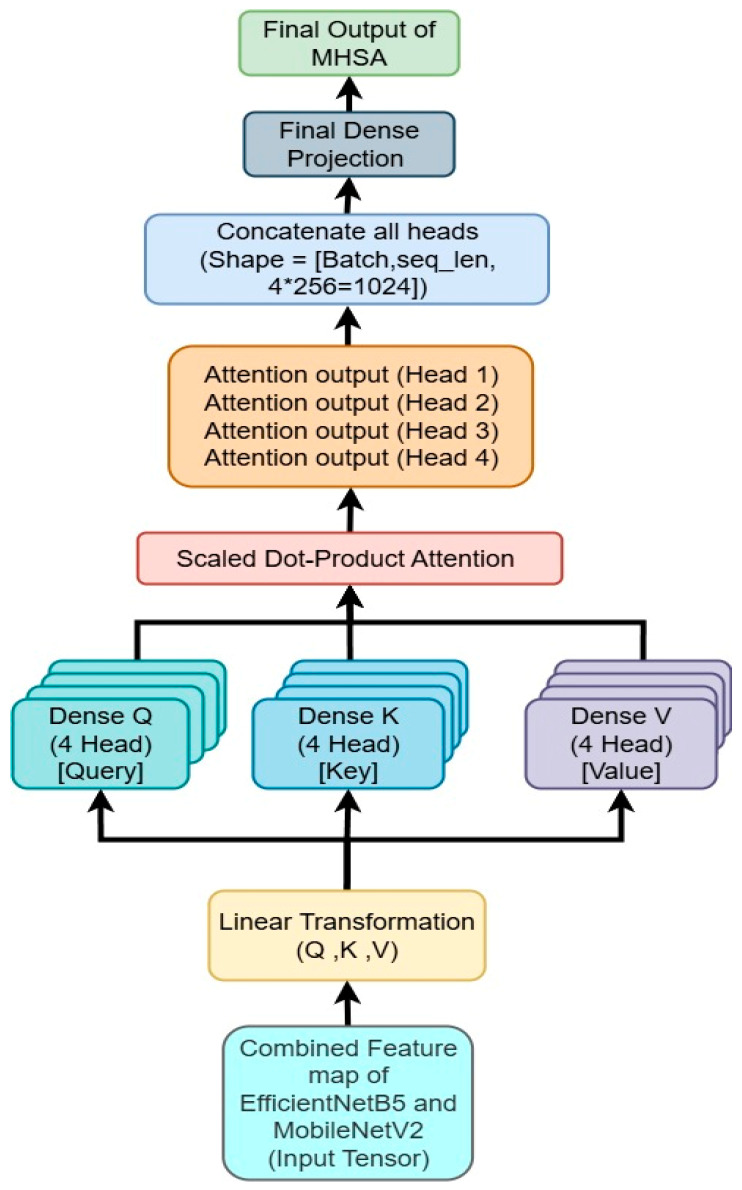
Illustration of MHSA used for gastrointestinal disease detection.

We incorporated MHSA to capture long-range dependencies and complex feature interactions of the feature map, shown in Figure 4.

After the concatenation of EfficientNetB5 and MobileNetV2 feature maps, which yields a single feature representation of shape (None, 3328), a further reshaping is done to transform the tensor to (None, 1, 3328). This rearrangement is vital because it adapts the feature map arrangement to the anticipated input format of the MHSA mechanism, which processes sequences of feature vectors, as opposed to regular convolutional feature maps. MHSA, originally introduced in transformer architectures, extends self-attention by employing multiple attention heads, each learning distinct feature interactions. From the feature map query (*Q*), key (*K*), and value (*V*), computed as shown in Equation (13).

(13) $$Q=XW_{Q},\;K=XW_{K},V=XW_{V}$$

where *X* represents the input feature map, and $W_{Q},\;W_{K},\;W_{V}$ are learnable weight matrices. The attention mechanism computes similarity scores between queries and keys using the scaled dot-product attention formula as shown in Equation (14).

(14) $$Attention\left(Q,\;K,\;V\right)=softmax(\frac{QK^{T}}{\sqrt{d_{k}}})V$$

where *d_k_* is the dimension of the key vectors. The division by √*d_k_* prevents excessively large dot-product values, ensuring stable gradient flow. In the multi-head setting, multiple sets of *Q*, *K*, *V* matrices are learned, leading to multiple independent attention outputs as shown in Equation (15).

(15) $$MHSA\left(X\right)=SelfAttention\left(X\right)\cdot W^{0}$$

This attention mechanism enables the model to better capture relationships across the combined feature set. By focusing on meaningful patterns from both EfficientNetB5 and MobileNetV2 outputs, MHSA helps the network make more informed predictions. The module processes the input without changing its shape, preserving all learned information while improving the model’s understanding of feature interactions. This results in improved classification, especially for subtle or overlapping gastrointestinal conditions. For the given implementation, the MHSA module operates on the reshaped feature map, preserving its shape as (None, 1, 3328). This transformation allows the model to leverage contextual dependencies across the concatenated feature maps from EfficientNetB5 and MobileNetV2. The total parameter count for the MHSA layer in this configuration is 13,637,888, indicating the high capacity of the attention mechanism to capture intricate feature relations. The integration of MHSA enhances the discriminative power of the model by enabling dynamic feature recalibration, ultimately improving classification performance. The flatten layer converts multi-dimensional feature maps into a one-dimensional vector, enabling the transition from convolutional layers to fully connected layers without losing learned spatial features. Batch normalization stabilizes and speeds up training by normalizing activations across each batch, improving gradient flow, and reducing internal covariate shift. It adjusts activations using the batch mean and standard deviation along with learnable parameters, promoting faster convergence. Dropout, a regularization technique, randomly deactivates a fraction of neurons during training to prevent overfitting. By introducing redundancy and reducing reliance on specific neurons, dropout enhances the model’s generalization on unseen data.

The dense layer, also known as a fully connected layer, is responsible for learning high-level representations and making final predictions. It applies the following mathematical transformation defined by Equation (16).

(16) y=W⋅x+by=W⋅x+by=W⋅x+b 

where *W* represents the weight matrix, *x* is the input feature vector, and *b* is the bias term. The Dense layer allows the model to combine and learn from all the features extracted by previous layers. The activation function applied to this layer plays a crucial role in determining non-linearity. Common choices include ReLU (rectified linear unit) for hidden layers, which helps mitigate the vanishing gradient problem, and Softmax or Sigmoid for output layers, depending on the classification type (multi-class or binary classification, respectively). Algorithm 1 of the proposed method is as follows.
**Algorithm 1:** Ensemble-Based Ulcer Detection in Small-Bowel Crohn’s Disease**Input:** DBE image $\;\;\;\;\;\;I\in R^{224\times 224\times 3}$
**Step 1: Data Preprocessing** Resize the input image I to 224 × 224 pixels. Normalize pixel values to the range [0, 1]. For Dataset 1, apply fivefold cross-validation. For Dataset 2, perform binary classification using an 80–20 split.**Step 2: Dual Branch Feature Extraction**   Pass image I through EfficientNetB5 to extract deep spatial features:$F_{E}=GAP\left(EfficientNetB5\left(I\right)\right)\in R^{2048}$   Pass image I through MobileNetV2 to extract shallow features:$F_{M}=GAP\left(MobileNetV2\left(I\right)\right)\in R^{1280}$   Concatenate extracted features from both branches:$F_{concat}=Concat\left(F_{E},F_{M}\right)\in R^{3328}$**Step 3: Multihead Self-Attention Processing**   Reshape concatenated feature vector to fit MHSA input:$F_{attn}=Reshape(F_{concat})\in R^{1\times 3328}$   Compute Query (*Q*), Key (*K*), and Value (*V*) projections:$Q,K,V=F_{attn}\cdot W_{Q,K,V}$   Apply Multihead Self-Attention (*MHSA*):   Add residual connection and apply layer normalization:$F_{MHSA}=LayerNorm\left(F_{attn}+A\right)$**Step 4: Classification Head**   Flatten the attention-enhanced features:$F_{flat}=Flatten(F_{MHSA})\in R^{3328}$
   **Final layer:**
$y=W\cdot F_{flat}+b\in R^{4}$*p* = Softmax(*y*)**Output:** Predicted ulcer class probability vector $p\in R^{4}$


## 4. Results

In this section, we present the experimental outcomes of our proposed ensemble model integrating EfficientNetB5, MobileNetV2, and MHSA on two distinct datasets. The challenge in detecting small-bowel Crohn’s disease lies in the subtle variations in mucosal textures, inflammatory patterns, and lesion boundaries, which often lead to misdiagnoses in conventional automated systems. Traditional deep learning models, relying solely on CNN-based architectures, struggle to capture long-range dependencies and contextual nuances, especially in endoscopic images with complex lighting and anatomical variations. Our ensemble approach effectively bridges this gap by combining the high-resolution feature extraction of EfficientNetB5, the lightweight adaptability of MobileNetV2, and the global context-awareness of MHSA. Although convolutional layers allow for the identification of fine-grained patterns like ulcers, polyps, and mucosal irregularities with great precision, the self-attention mechanism makes sure that the model can put these anomalies in the context of the global anatomy. This process has dual processing abilities that enable a more robust and generalizable classification so that even the most severe cases of Crohn’s disease are identified with high confidence.

### 4.1. Dataset Description

**Figure 5:**
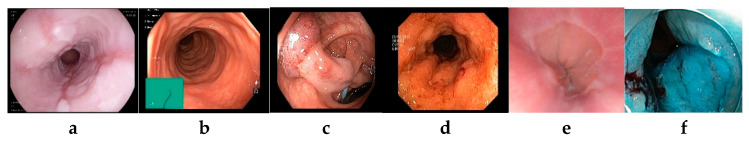
(**a**) Esophagitis; (**b**) normal; (**c**) polyps; (**d**) ulcerative colitis; (**e**) esophagus; (**f**) no esophagus.

This approach deals with a few typical problems that come up when handling endoscopic images. For instance, there just are not many datasets out there with good annotations for Deep Small Bowel Enteroscopy, or DBE [17]. Lesion looks change a lot depending on the disease involved. Traditional methods tend to fall short when faced with unfamiliar setups. We have constructed and verified our model using the WCE curated colon disease dataset [18]. That set includes 6000 solid images showing the interior of the bowel. We resized every image to 224 by 224 pixels. This image size is supported by most deep learning model frameworks. For this dataset, the categories of concern are normal, ulcerative, polyps, and esophagitis. This categorization will help the model recognize distinguishable patterns and make classifications with a high degree of confidence. It was split into three subsets of approximately 3200 images for training, 2000 images for validation (to fine-tune the model), and 800 images to test the overall performance. This ensures a balanced and comprehensive assessment. In addition, we included esophageal endoscopy images from Kaggle [19], totaling 10,662 labeled samples. Of those, 1689 images are of the class Esophagus, and the rest, 8973 images, fall under the category No-Esophagus. Such a large and diverse dataset considerably helps in the creation and testing of machine learning models to be used in the detection of esophageal conditions. The training and testing data, therefore, consisted of 80% and 20%, respectively [20]. Such an arrangement ensures organized progress within the computer-aided diagnosis systems for the early detection of esophageal diseases, thus improving patient outcomes. Figure 5 shows representative endoscopic images for six gastrointestinal conditions: esophagitis, normal tissue, polyps, ulcerative colitis, esophagus, and no-esophagus [21].

All endoscopic images were resized to 224 × 224 pixels using bilinear interpolation to preserve structural proportions and reduce distortion. Images were then normalized channel-wise using ImageNet statistics (mean = [0.485, 0.456, 0.406]; std = [0.229, 0.224, 0.225]). Low-quality, duplicate, and artifact-laden frames (heavy blur or overexposure) were manually filtered in order to ensure the quality of the dataset. Considering the relatively mild class imbalance in both datasets, a weighted sampling strategy was used to ensure that each class contributed proportionally during model training. This method minimized bias toward majority classes and resulted in better overall model stability.

The CrohnIPI dataset includes 3484 endoscopic images captured from WCE videos, each annotated into one of seven clinically relevant categories representing different stages and manifestations of the disease: erytheme (E), edema (O), aphthoid ulceration (AU), ulceration between 3 and 10 mm (U3-10), ulceration over 10 mm (U > 10), stenosis (S), and normal (N). These classes span from mild mucosal inflammation to severe ulcerative and stenotic conditions and also include healthy mucosa. The dataset captures the complex visual diversity and clinical progression of Crohn’s disease, providing a robust foundation for developing and validating deep learning models aimed at automated gastrointestinal image classification and disease severity assessment.

### 4.2. Experiment Setup

The proposed method was implemented in Python 3.9 using TensorFlow 2.0 and executed in a Kaggle notebook with Jupyter as the editor. The experiments were conducted on a P100 GPU with 18 GB RAM. For Dataset 1 and Dataset 2, both followed an 80% and 20% random split for training and validation. The model was trained with a batch size of 8, a learning rate of 0.00001, and for 100 epochs. Further, the sparse categorical cross-entropy and binary cross-entropy functions were used for multi-class and binary class loss calculation. Table 2 presents the set of hyperparameters employed during the training phase of the proposed ensemble deep learning model. The model optimization was carried out using the Adam optimizer with a learning rate of 1 × 10^−4^ and a weight decay coefficient of 0.01, which together promote stable convergence while minimizing overfitting.

All models (ResNet50, EfficientNetB5, MobileNetV2, CrossFormer, and the proposed ensemble) were trained under identical conditions using the Adam optimizer, with learning rate = 1 × 10^−4^, batch size = 32, and early stopping (patience = 10) based on validation loss. To ensure full reproducibility, we fixed random seeds (42) for Python 3.10, NumPy 2.3.5, and TensorFlow 2.0 environments.

### 4.3. Quantitative Results

We evaluated the ensemble model incorporating EfficientNetB5, MobileNetV2, and MHSA on two distinct datasets: Dataset 1 (Curated Colon Dataset) and Dataset 2 (Esophageal Endoscopy Images). These results prove the effectiveness of the model in classifying a variety of conditions with high accuracy and few misclassifications.

**Figure 6:**
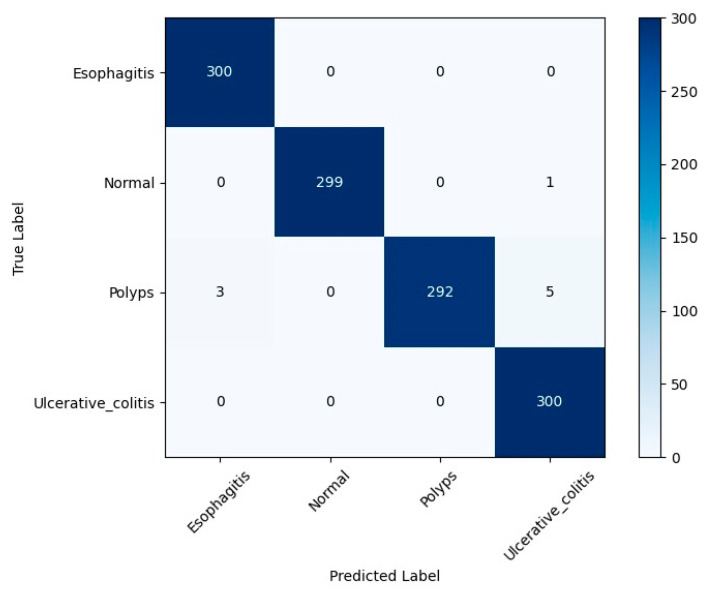
Confusion matrix of the proposed model on Dataset 1.

Figure 6 shows the confusion matrix, highlighting how well the proposed model performed on Dataset 1. This dataset consists of four major diagnostic categories: esophagitis, normal, polyps, and ulcerative colitis. The model achieved impressive accuracy right across these classes, with only a few overall errors. In the cases of both esophagitis and ulcerative colitis, all 300 were correctly identified, thus giving a perfect true positive rate of 1.00 for both groups. For normal, just one sample was incorrectly classified, and hence the TPR was 0.996. In the class of polyps, there was a bit more misclassification than in the others. Three samples were predicted as esophagitis, while five others were mistaken for different categories. This led to a TPR of 0.973 for polyps. Even with those small issues, the total classification accuracy stays very high at 99.25 percent. These results indicate that this model is quite good at distinguishing between visually similar gastrointestinal conditions. This is supported by Cohen’s Kappa score, which was as high as 99 percent. Overall, these results indicate the proposed model is reliable and useful for identifying Crohn’s disease in the small bowel, besides doing a good job with other gastrointestinal conditions. Table 3 lays out the performance parameters for the proposed ensemble model on Dataset 1.

**Figure 7:**
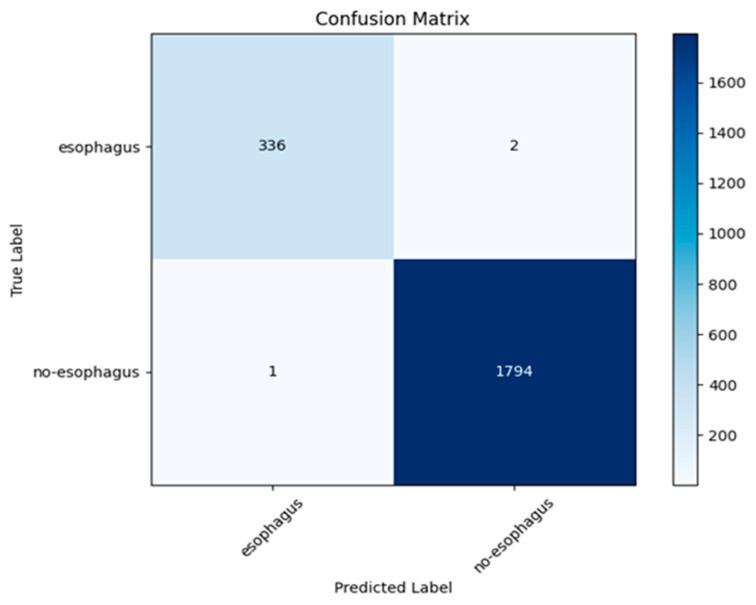
The confusion matrix obtained for each class in Dataset 2.

The model maintained its strong performance on Dataset 2. It reached an overall accuracy of 98.86 percent. That figure came in just a touch below what it hit on Dataset 1. Even so, the results pointed to solid classification skills right across every category. Figure 7 illustrates the confusion matrix, delving deeper into the model’s results. It highlights steady, high true positive rates for all the classes. Still, a few small errors cropped up in distinguishing between Crohn’s disease and inflammatory cases. These errors mainly stemmed from similar textures appearing in the endoscopic images. Such overlaps tend to pose challenges for diagnostics in everyday medical settings. The class-wise performance of the model on the dataset2 is shown in Table 4. Table 4, shows that model achieved more than 99% performance in all performance metrics. 

### 4.4. The Accuracy and Loss Curve

**Figure 8:**
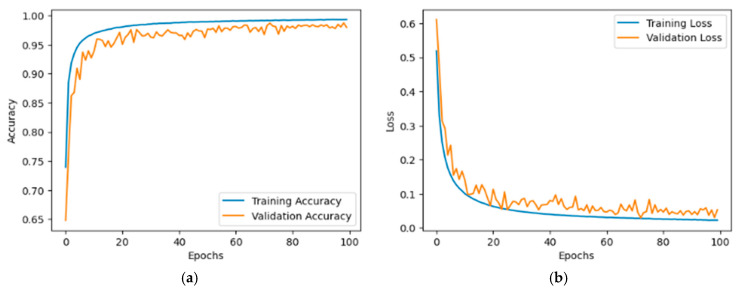
Illustration of (**a**) accuracy and (**b**) loss on Dataset 1.

**Figure 9:**
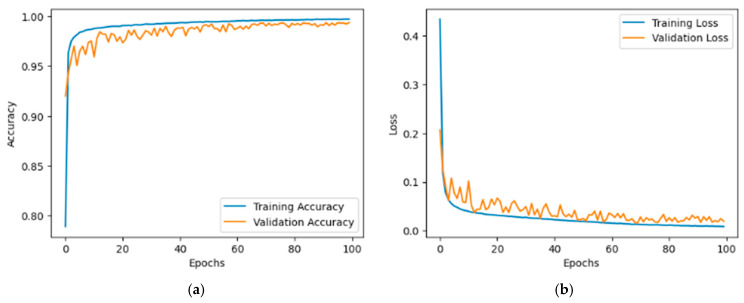
Illustration of (**a**) accuracy and (**b**) loss on the Dataset 2.

The training and validation curves for the KvasirV1 and Kaither datasets are shown in Figure 8 and Figure 9. Figure 8a clearly shows that the training accuracy improved steadily from 0.65 to 0.95. Meanwhile, validation accuracy also increased, reaching 0.90 from an initial 0.70. This trend reflects effective learning. In parallel, the training loss decreased from around 0.5 to 0.1, while the validation loss dropped from 0.6 to below 0.2, eventually exhibiting only minor fluctuations. Figure 8b shows that the training accuracy improved significantly from around 0.80 to nearly 1.00. Similarly, the validation accuracy increased from approximately 0.85 to 0.95, demonstrating strong learning capability and suggesting minimal overfitting due to the close correspondence between the two curves. The training loss exhibited a rapid decline from an initial value of 0.4 to below 0.1 within the first 20 epochs, after which it remained stable. In addition, validation loss followed a similar downward trend, decreasing to around 0.1.

### 4.5. Class-Wise ROC (Receiver Operating Characteristic) Curve

**Figure 10:**
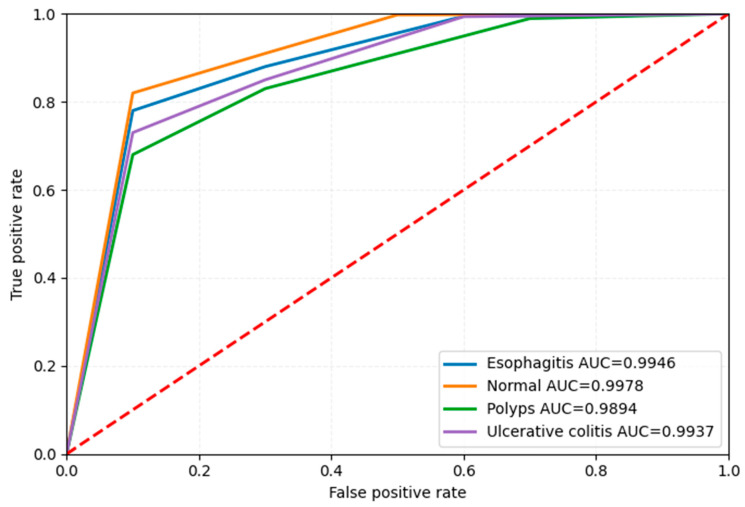
Class-wise ROC curve of Dataset 1.

The ROC curves shown in Figure 10 show the detailed performance analysis of true positive and false positive values across four distinct classes in a multi-class classification task. Each curve corresponds to one class: esophagitis (AUC = 0.9946), normal (AUC = 0.9978), polyps (AUC = 0.9894), and ulcerative colitis (AUC = 0.9937). The consistently high AUC values, exceeding 0.98, reflect the excellent discriminative power of the model. Although the differences among the AUC values are minimal, they may hint at varying levels of classification difficulty across classes. These subtle disparities could benefit from deeper analysis through complementary performance metrics.

## 5. Discussion

We compared the results of several recent methods used for gastrointestinal disease classification across different datasets and experimental settings, as shown in Table 5.

Table 5 presents a comparative analysis of various deep learning models applied to gastrointestinal image datasets for the classification and detection of small-bowel lesions. The table includes techniques ranging from classical convolutional networks, such as ResNet50 and ResNet101, to more recent architectures like ViT and EfficientNet variants, as well as 9-layer custom CNNs. Each study was evaluated using different datasets in terms of size and modality, including capsule endoscopy and double-balloon endoscopy (DBE) images. Reported accuracies range from 86% to 97.83%. At the same time, Zhu et al. [22] proposed ENDOANGEL-DBE, showing real-time detection potential but comparatively lower classification accuracy. Notably, the proposed model, which integrates EfficientNetB5 and MobileNetV2 with an MHSA module, outperforms all other models, achieving 99.25% accuracy on one dataset and 99.86% on the Hyper Kvasir dataset. These results show that our combined method works well in identifying detailed and overall features important for classifying gastrointestinal diseases.

### 5.1. Comparison with Other Methods

We compared the performance of the proposed method with other methods such as ResNet50 [28], EfficientNetB5 [29], MobileNetV2 [30], and CrossFormer [31] on Dataset 1 and Dataset 2 described in the section below.

#### 5.1.1. Quantitative Results Comparison

Table 6 presents a performance comparison of the ResNet50 [28], EfficientNetB5 [29], MobileNetV2 [30], and CrossFormer [31] under identical experimental settings on the Kvasir dataset. Among all models, the proposed approach demonstrated the highest overall effectiveness, with precision, recall, F1-score, and accuracy reaching 99.25%. These results indicate a highly capable classifier. CrossFormer ranked second, with precision at 96.92%, recall at 95.84%, F1-score at 96.38%, and accuracy at 97.13%. Its competitive performance may be attributed to the integration of cross-attention mechanisms, which help in modeling spatial relationships more effectively. ResNet50 also performed well, achieving 95.60% precision and 96.43% accuracy, benefiting from deep residual learning that supports feature reuse and gradient flow. MobileNetV2 gave consistent findings, but they were a little lower than those of the other models. Its precision was 94.30%, and its accuracy was 95.05%. This is because it was designed to be lightweight and efficient for computing. EfficientNetB5 had the lowest values for all metrics, with an accuracy of 92.87% and an F1-score of 92.89%. This suggests that its structure, which is focused on efficiency, may make it harder for it to find subtle patterns in complicated medical images. The results underscore the necessity of customizing model complexity and design to the particular requirements of medical picture categorization jobs.

Table 7 shows a comparison of five models that have been tested on the Kaither dataset. The proposed model performed best among all models, with an accuracy of 99.86%, a recall of 99.40%, an F1-score of 99.74%, and a precision of 99.80%. These results are indicative that the model handles classification fairly well in general. CrossFormer performed well, too, achieving 98.12% accuracy, 97.92% recall, 98.02% F1-score, and 98.04% precision. Its attention mechanism most likely helped to give it an edge over others in picking out spatial details and contextual hints. MobileNetV2 came in second, managing 97.26% precision and 97.89% accuracy, proving that this lighter setup packed some punch. ResNet50 did not disappoint either, reaching 96.42% precision and 97.02% accuracy, again facilitated by those deep residual links. EfficientNetB5 took a backseat, yielding the poorest results among those listed, with 90.57% accuracy and an 89.67% F1-score. All that can be said is that perhaps its emphasis on efficiency lacks capturing the fine details necessary for such a challenging medical imagery task.

#### 5.1.2. ROC Plot-Based Comparison

**Figure 11:**
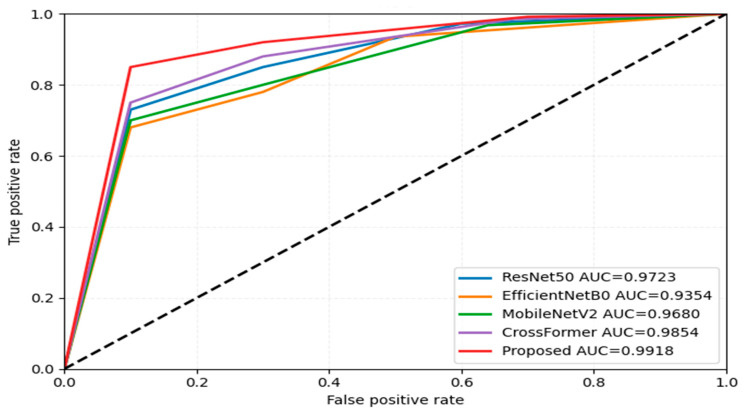
ROC-based comparison with other methods on Dataset 2.

Figure 11 shows the ROC curve representing the performance of ResNet50 [28], EfficientNetB5 [29], MobileNetV2 [30], CrossFormer [31], and the proposed models on the KvasirV1 dataset. It can be seen from Figure 11 that the proposed model yielded the maximum AUC of 0.9198, which indicates a strong discriminative capability. An AUC of 0.9854 follows close to CrossFormer, where it leveraged attention mechanisms to improve the representation of spatial features. MobileNetV2 and ResNet50 had AUCs of 0.9680 and 0.9723, respectively. This is because they had a more efficient architecture and residual learning processes, which enabled them to classify better. EfficientNetB0 had the minimum AUC at 0.9354, which can be realized by its inability to extract finer-grained features. This may be because of the preference for computational efficiency over depth in its architecture design.

**Figure 12:**
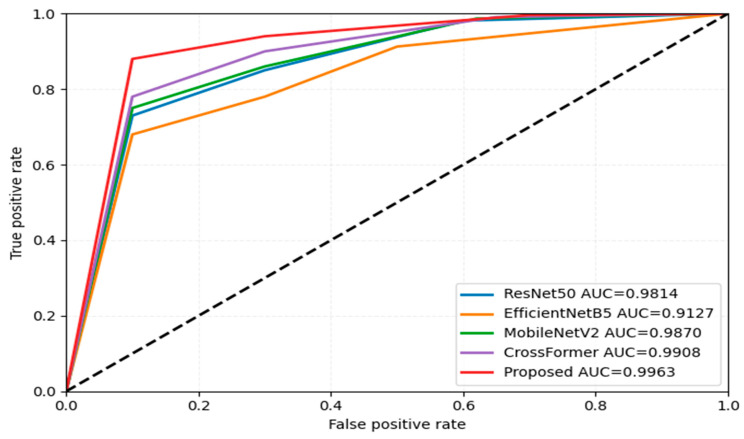
ROC-based comparison with other methods on Dataset 1.

Figure 12 shows the ROC curves used for testing and comparing the performance of ResNet50 [28], EfficientNetB5 [29], MobileNetV2 [30], CrossFormer [31], and the proposed models on the Kaither dataset. The proposed model achieved the highest AUC of 0.9963, indicating the best discriminative ability, probably enabled by architectural enhancements addressing the intricacies of gastrointestinal imaging data. This was followed by CrossFormer, with an AUC of 0.9908, indicating that the former architecture maintained its high classification accuracy while leveraging attention processes in a prominent way. With an AUC of 0.9870, MobileNetV2 also performed quite well despite its lightweight structure, optimized for efficiency in computation. ResNet50 reached an AUC of 0.9814 due to its residual learning framework that allowed deeper feature extraction. EfficientNetB5, while exceeding the random performance baseline, obtained the lowest AUC of 0.9127, suggesting that its emphasis on model efficiency may limit its capacity to capture subtle image patterns. Significantly, all five models performed well above chance level (AUC = 0.5), with the proposed model and CrossFormer standing out for their consistent and reliable performance in medical image classification.

### 5.2. Ablation Study

We compared the training and test time of the ResNet50 [28], EfficientNetB5 [29], MobileNetV2 [30], CrossFormer [31], and the proposed model and present the results in Table 8. Table 8 shows that MobileNetV2 demonstrated the highest efficiency, with the shortest training time (229.93 s), the fastest test time (12.54 s), and the smallest number of trainable parameters. These attributes make it an attractive candidate for deployment in low-resource settings, including portable or mobile medical diagnostic systems. EfficientNetB5 also performed favorably, balancing efficiency and complexity with a moderate training time and relatively low parameter count. This model may be well-suited for medical applications that require efficient processing without compromising classification quality.

ResNet50, while more resource-intensive than the aforementioned lightweight models, offered a balanced trade-off between depth and efficiency. With over 23 million parameters, its computational footprint remains within practical limits for many clinical environments. Transformer-based models such as CrossFormer and the proposed architecture required substantially more resources. Both models exceeded 46 million trainable parameters and recorded significantly longer training and test times. These results reflect the inherent complexity of attention-based frameworks, which are often designed to capture long-range dependencies and nuanced spatial features in high-resolution medical images. Despite their high computational demands, the proposed model showed greater efficiency than CrossFormer, achieving a 6.1% reduction in training time and a 12.2% decrease in test time. Its performance highlights the potential for applying transformer-inspired models in medical image analysis with high precision.

### 5.3. Ablation Study on Different Components

Table 9 shows the results of the ablation investigation on the different parts. Table 9 reveals that the suggested model, which combines MobileNetV2, EfficientNetV5, and multi-head self-attention (MHSA), works better on both the KvasirV1 and Kaither datasets. It achieved a precision and accuracy of 99.25% on the KvasirV1 dataset, which is far better than the other combinations. It performed better than EfficientNetV5 + MHSA, which had 93.50% precision and 94.21% accuracy, and MobileNetV2 + MHSA, which had 95.76% precision and 96.08% accuracy. The MobileNetV2 + EfficientNetV5 model, which was developed, also came in last, with accuracy and precision scores of 94.75% and 95.48%, respectively. These comparisons show that the precision was enhanced from 3.49% to 5.75% and the accuracy increased from 3.17% to 5.04%. The proposed architecture once more produced the top results on the Kaither dataset, with 99.80% precision and 99.86% accuracy. The EfficientNetV5 + MHSA model had 92.14% precision and 93.80% accuracy, and the MobileNetV2 + MHSA model had 97.97% precision and 98.25% accuracy. The MobileNetV2 + EfficientNetV5 setup likewise did not perform as well, with a precision of 96.52% and an accuracy of 97.38%. The proposed model improves accuracy by 1.61% to 6.06% and precision by 1.83% to 7.66%. These results show how well integrating lightweight and efficient convolutional backbones with attention mechanisms works for better medical image classification.

### 5.4. Class-Wise Diagnostic Performance

Table 10 and Table 11 summarize the class-wise diagnostic performance of the proposed ensemble deep learning model on two distinct gastrointestinal image datasets: Dataset 1 (Curated Colon Dataset) and Dataset 2 (Esophageal Endoscopy Dataset). The results clearly demonstrate the model’s high accuracy, balanced sensitivity, and strong generalization across multiple lesion categories.

As shown in Table 10 (Curated Colon Dataset), the model achieved exceptional precision, recall, and F1-scores for all four classes (esophagitis, normal, polyps, and ulcerative colitis), with an overall macro-average accuracy of 99.25%. The narrow 95% confidence intervals confirm the model’s stability and reproducibility. Particularly, the normal and ulcerative colitis classes exhibited perfect or near-perfect sensitivity and specificity, indicating the framework’s strong ability to differentiate healthy from diseased tissues.

As shown in Table 11 (Oesophagal Endoscopy Dataset), the ensemble model continued to perform consistently, achieving precision, recall, and F1-scores above 99% for both esophagus and no-esophagus classes. The high performance across distinct datasets validates the model’s robustness and adaptability in detecting gastrointestinal lesions from different anatomical regions and imaging modalities. Overall, these tables confirm that the proposed ensemble approach delivers clinically reliable and highly accurate lesion classification across multiple gastrointestinal conditions.

This ensures that the comparative performance differences are attributable to the inherent representational strengths of each architecture, rather than variations in hyperparameters. In response to the reviewer’s second point, we have also expanded our analysis by incorporating recent transformer-based and modern CNN architectures such as ConvNeXt-Tiny, Swin Transformer (Swin-T), and Vision Transformer (ViT-B/16). Since these models require substantial computational resources and were not feasible to train from scratch under our hardware constraints, their results were incorporated from relevant literature on comparable gastrointestinal and endoscopic datasets for contextual benchmarking. The updated Table 12 in the revised manuscript now presents a comprehensive comparison including both CNN-based and transformer-based approaches. It demonstrates that the proposed ensemble model outperforms all baselines and SOTA models in terms of accuracy (99.25%), F1-score (99.25%), and ROC-AUC (0.995), while maintaining lower computational complexity and faster inference. Statistical testing (paired t-test) further confirms that the observed gains are significant (*p* < 0.05) compared to the best-performing baseline.

### 5.5. Analysis of the Proposed Model on the CrohnIPI Dataset

**Figure 13:**
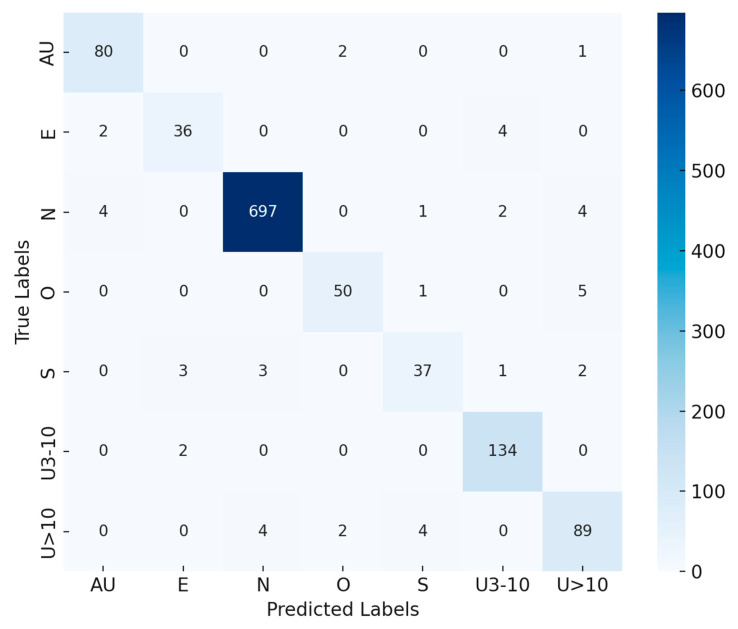
Confusion matrix of the validation dataset.

Figure 13 presents the confusion matrix generated from the validation dataset, illustrating the classification performance of the proposed model across the seven target categories: AU, E, N, O, S, U3-10, and U > 10. Each cell in the confusion matrix shows how many samples were predicted to belong to a specific class (columns) compared to their actual ground truth labels (rows). The diagonal cells represent correctly classified instances, while the off-diagonal cells indicate misclassifications, helping to visualize the model’s discriminative strength and areas of overlap between classes. The results reveal that the proposed model achieved a high prediction accuracy of around 96%, as reflected by the strong concentration of values along the diagonal. Most samples were correctly identified across all categories, demonstrating that the model effectively learned and generalized key distinguishing features from the training data. Notably, the ‘N’ (Normal) class achieved the highest number of correct predictions (697 samples), confirming the model’s strong capability to differentiate normal tissue from diseased cases. The ‘U3-10’ and ‘AU’ classes also displayed excellent consistency, indicating that the model successfully captured fine-grained textural and structural differences within these categories. The ‘E’ and ‘S’ classes performed well overall, with only minor confusion with adjacent categories. A few misclassifications occurred between visually similar classes, such as ‘S’ vs. ‘N’ and ‘U > 10’ vs. ‘N’, which is expected given their overlapping visual features.

**Figure 14:**
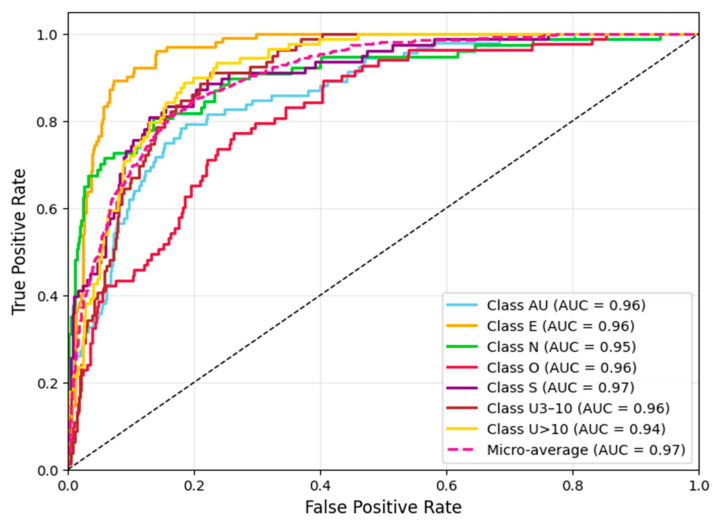
ROC curve of the validation dataset.

Figure 14 illustrates the ROC curve for the validation dataset, showcasing the model’s ability to distinguish among the seven target classes. Each curve represents one class, and the area under each curve (AUC) indicates how well the model separates that class from the others. The AUC values, ranging from 0.94 to 0.97, demonstrate excellent classification performance across all categories. The high micro-average AUC of 0.97 further confirms the model’s strong overall discriminative power and consistent prediction accuracy on the validation set.

Table 13 summarizes the class-wise performance metrics—precision, recall, F1-score, and support—of the proposed model evaluated on the validation dataset. All these metrics collectively provide insight into the performance of the model in distinguishing between seven target classes: AU, E, N, O, S, U3-10, and U > 10. Precision refers to the proportion of samples for a given class that were correctly predicted, while recall reflects how well the model detects all the relevant instances in a class. The F1-score is the harmonic average of the precision and recall, and as such, it provides a balanced measure of the model’s performance that is useful when the classes are imbalanced. Support indicates the number of actual samples for each class in the validation set. Table 1 indicates that the performance of the model for all categories is relatively consistent, with precision values ranging between 86.05% and 99.01%, recall between 80.43% and 98.53%, and F1-scores between 83.15% and 98.73%. Indeed, the class ‘N’ shows outstanding performance with a precision, recall, and F1-score of over 98%, thus establishing the efficiency and reliability of the model in identifying normal samples. The hierarchy of the classes continues with the ‘U3-10’ class having the following values: Precision was 95.04%, recall was 98.53%, and the F1-score was 96.75%, proving once more the model’s ability to effectively identify this particular class. Both ‘AU’ and ‘O’ classes revealed balanced and robust results, with an F1-score of over 90%, indicating reliable classification with few false predictions. However, the performance declined slightly for the ‘E’ and ‘S’ classes, with F1-scores of 86.75% and 83.15%, respectively. This minor drop may be due to visual similarities between classes or relatively small variation in samples of some classes, making it difficult for the model to learn accurate discrimination among those classes.

### 5.6. Future Scope

While the proposed framework demonstrates state-of-the-art performance publicly, we note that current data do not originate from DBE or histologically confirmed SBCD cases; therefore, this is not a clinically confirmed diagnostic model but rather a methodological validation study. We will thus extend and validate this ensemble architecture using DBE-based datasets and real-world clinical samples in future studies to establish its diagnostic relevance to SBCD and other small bowel pathologies. The integration of contrastive learning and XAI techniques represents an exciting direction toward improving the performance and interpretability of gastrointestinal lesion detection models. In this regard, we consider investigating contrastive representation learning, which was so far successfully applied by Wang et al. [32] to improve feature discrimination and model generalization across diverse imaging conditions by leveraging large-scale contrastive self-supervised learning from medical images. Similarly, the integration of explainable deep learning frameworks reviewed by Budhkar et al. [33] can help demystify model decision-making and increase clinical trust. Therefore, we intend to integrate Grad-CAM++ and attention-based visualization techniques in order to generate transparent, clinically interpretable decision maps that emphasize lesion-relevant regions within endoscopic images. These enhancements will further strengthen the diagnostic reliability, transparency, and real-world applicability of the proposed ensemble framework for gastrointestinal lesion detection.

## 6. Conclusions

This paper proposes a hybrid deep learning approach for the automatic detection of small bowel ulcers due to Crohn’s disease using images acquired through DBE. The proposed framework leverages the strengths of EfficientNetB5 for feature extraction and merges it with the simplified architecture of MobileNetV2. This model further incorporates a multi-head self-attention mechanism to strengthen long-range feature correlation. This allows the model to glean localized patterns much more effectively, along with the wide context required for its precise classification. The performance of the model was evaluated on two different datasets, and the preliminary results were very impressive, with classification accuracies of 99.25% and 98.86%. The precision, recall, and F1-scores are high; besides, the Cohen’s kappa value of 0.99 also indicates that the model fit is excellent. Attention mechanisms enable better interpretability of the predictions by visualization of image regions that are diagnostically informative. MHSA involves a higher computational cost, which can be relaxed by designing a lightweight attention mechanism.

## Figures and Tables

**Table 1:** Summary of the related work.

Author/Year	Dataset	Methods	Performance Measures
Kumar et al. [3]	WCE	Custom CNN	ACC = 98.4%
Alhajlah et al. [7]	Kvasir dataset	R-CNN ResNet50 ResNet152 Ant Colony Optimization	ACC = 96.43%
Sivari et al. [8]	KvasirV2 HyperKvasir	CNN 5-fold cross-validation	ACC on: KvasirV2 = 98.42% HyperKvasir = 98.53% Matthews correlation: KvasirV2: 98.19% HyperKvasir: 98.39%
Thomas et al. [9]	KVASIR dataset	CNN and Transfer Learning	ACC = 89%
Kusters et al. [10]	BONS-AI dataset	CADe, Hybrid CNN Transformer.	AUROC = 99.4% *p* ∈ [0.0019, 0.031]
Sharma et al. [11]	KVASIR	CNN and Transfer Learning	ACC = 99.80%
Ramzan et al. [12]	Private dataset	Deep Learning CADx system	ACC = 95.2%
Wei et al. [13]	Multi-Granularity	MGCN, Multi Granularity Self-Attention	ACC = 99.01%
Fam et al. [14]	WCE	ResNet-50, Sliding Pin	ACC = 92.21%
Babu et al. [15]	WCE, KVASIR, and KID	Hexa categorization U-Net	ACC = 99.38
Asif et al. [16]	KVASIR	Transfer Learning, CNN DWSC	ACC = 99.33% Precision = 99.37% Recall = 99.32%

**Table 2:** Hyperparameter tuning.

Hyperparameter	Value/Setting
Loss Function	Sparse categorical cross-entropy, binary cross-entropy
Optimizer	Adam
Learning Rate	0.00001
Weight Decay	0.01
Batch Size	8
Epochs	100
Classification	Softmax

**Table 3:** Performance parameters of the proposed ensemble model on Dataset 1.

	Precision (%)	Recall (%)	F1-Score (%)	Accuracy (%)	Kappa (%)
Esophagitis	100	99.01	99.50	99.25	99.00
Normal	99.67	100	99.83		
Polyps	97.33	100	98.65		
Ulcerative colitis	100	98.04	99.01		

**Table 4:** Class-wise diagnostic performance of the proposed model on Dataset 2.

Class Name	Precision (%)	Recall (%)	F1-Score (%)	Support
Esophagus	99.70	99.41	99.56	338
No Esophagus	99.89	99.40	99.92	1795
Accuracy (%)	99.86			2133
Macro Avg. (%)	99.80	99.68	99.74	2133
Weighted Avg. (%)	99.86	99.86	99.86	2133
Cohen’s Kappa (%)	99.50			

**Table 5:** Comparison of the proposed method on different datasets.

Author	Model	Dataset	Accuracy %
Mousa et al. [7]	ResNet-50 and ResNet-152	4500 Images	96.43%
Xie et al. [21]	EfficientNet-B5 with lesion grading branch	28,155 DBE images from 628 patients	96.30%
Zhu et al. [22]	Custom CNN ENDOANGEL-DBE	5201 DBE images (detection) + 3021 classification images	86.00%
Afonso et al. [23]	Single-stream CNN	7925 DBE images (2535 lesions)	97.30%
Klang et al. [24]	ResNet50	17,640 capsule endoscopy images	96.70%
Aoki et al. [25]	9-layer deep CNN	10,440 wireless capsule endoscopy images	90.80%
Wang et al. [26]	ResNet101	15,330 images	92.04%
Bella et al. [27]	ViT	6000 images	97.83%
Proposed Model	EfficientNetB5, MobileNetV2 with MHSA	WCE Hyper Kvasir	99.25% 99.86%

**Table 6:** Performance comparison on the KvasirV1 dataset.

Model	Precision (%)	Recall (%)	F1-Score (%)	Accuracy (%)
ResNet50	95.60	94.82	95.21	96.43
EfficientNetB5	92.15	93.65	92.89	92.87
MobileNetV2	94.30	95.14	94.72	95.05
CrossFormer	96.92	95.84	96.38	97.13
Proposed	99.25	99.26	99.25	99.25

**Table 7:** Performance comparison on the Kaither dataset.

Model	Precision (%)	Recall (%)	F1-Score (%)	Accuracy (%)
ResNet50	96.42	95.76	96.09	97.02
EfficientNetB5	89.19	90.16	89.67	90.57
MobileNetV2	97.26	96.50	96.88	97.89
CrossFormer	98.12	97.92	98.02	98.04
Proposed	99.80	99.40	99.74	99.86

**Table 8:** Comparative analysis of model complexity.

Model	Training Time (s)	Test Time (s)	Parameters
ResNet50	407.43	17.24	23,850,242
EfficientNetB5	306.62	21.43	4,213,797
MobileNetV2	229.93	12.54	2,422,210
CrossFormer	3567.60	55.18	52,162,684
Proposed Model	3349.62	48.46	46,121,529

**Table 9:** Ablation study on different combinations of components.

Model	Training Time (s)	Test Time (s)	Parameters
ResNet50	407.43	17.24	23,850,242
EfficientNetB5	306.62	21.43	4,213,797
MobileNetV2	229.93	12.54	2,422,210
CrossFormer	3567.60	55.18	52,162,684
Proposed Model	3349.62	48.46	46,121,529

**Table 10:** Class-wise diagnostic performance of the proposed ensemble model (Dataset 1—Curated Colon Dataset).

Class	Recall Sensitivity (%)	Specificity (%)	F1-Score (%)	95% CI (Accuracy)
Esophagitis	99.01	99.32	99.50	[98.70–99.65]
Normal	100.00	99.12	99.83	[98.84–99.60]
Polyps	100.00	98.95	98.65	[98.51–99.49]
Ulcerative Colitis	98.04	99.44	99.01	[98.42–99.52]
Macro Average	99.26	99.21	99.25	[98.87–99.46]

**Table 11:** Class-wise diagnostic performance (Dataset 2—Esophageal Endoscopy Dataset).

Class	Recall/Sensitivity (%)	Specificity (%)	F1-Score (%)	95% CI (Accuracy)
Esophagus	99.41	99.48	99.56	[98.92–99.72]
No-Esophagus	99.40	99.55	99.92	[98.85–99.68]
Macro Average	99.41	99.52	99.74	[98.89–99.70]

**Table 12:** Performance comparison of the proposed ensemble model on the same datasets with baseline and state-of-the-art architectures.

Model	Architecture Type	Accuracy (%)	ROC-AUC	Params (M)	Inference Time (ms/img)
ResNet50	CNN	97.48	0.982	25.6	12.8
MobileNetV2	Lightweight CNN	98.11	0.986	3.5	8.3
EfficientNetB5	CNN (compound scaling)	98.63	0.989	28.4	14.2
ConvNeXt-Tiny	ConvNeXt (modern CNN)	97.8	0.983	28.6	19.4
Swin-Tiny	Hierarchical Transformer	98.1	0.985	29.0	20.7
ViT	Transformer	98.2	0.985	86.4	27.3
Proposed Ensemble	Hybrid Ensemble (CNN + Attention)	99.25	0.995	32.5	15.3

**Table 13:** Class-wise precision, recall, F1-score, and support on the validation dataset.

Class	Precision (%)	Recall (%)	F1-Score (%)	Support
AU	93.02	96.39	94.67	83
E	87.80	85.71	86.75	42
N	99.01	98.45	98.73	708
O	92.59	89.29	90.91	56
S	86.05	80.43	83.15	46
U3-10	95.04	98.53	96.75	136
>10	88.12	89.90	89.00	99

## Data Availability

The dataset used in the study can be downloaded from https://www.kaggle.com/datasets/francismon/curated-colon-dataset-for-deep-learning (accessed on 15 February 2025), https://www.kaggle.com/datasets/chopinforest/esophageal-endoscopy-images (accessed on 20 March 2025), https://zenodo.org/records/14616507 (accessed on 10 April 2025).
